# Activation of NF-κB and induction of proinflammatory cytokine expressions mediated by ORF7a protein of SARS-CoV-2

**DOI:** 10.1038/s41598-021-92941-2

**Published:** 2021-06-29

**Authors:** Chia-Ming Su, Leyi Wang, Dongwan Yoo

**Affiliations:** 1 Department of Pathobiology, University of Illinois at Urbana-Champaign, 2001 Lincoln Ave, Urbana, IL 61802 USA; 2 Department of Veterinary Diagnostic Laboratory and Department of Veterinary Clinical Medicine, University of Illinois at Urbana-Champaign, Urbana, IL USA

**Keywords:** SARS-CoV-2, Viral host response

## Abstract

Severe acute respiratory syndrome coronavirus 2 (SARS-CoV-2) is the causative agent for coronavirus disease 2019 (COVID-19) that emerged in human populations recently. Severely ill COVID-19 patients exhibit the elevation of proinflammatory cytokines, and such an unbalanced production of proinflammatory cytokines is linked to acute respiratory distress syndrome with high mortality in COVID-19 patients. Our study provides evidence that the ORF3a, M, ORF7a, and N proteins of SARS-CoV-2 were NF-κB activators. The viral sequence from infected zoo lions belonged to clade V, and a single mutation of G251V is found for ORF3a gene compared to all other clades. No significant functional difference was found for clade V ORF3a, indicating the NF-κB activation is conserved among COVID-19 variants. Of the four viral proteins, the ORF7a protein induced the NF-κB dictated proinflammatory cytokines including IL-1α, IL-1β, IL-6, IL-8, IL-10, TNF-α, and IFNβ. The ORF7a protein also induced IL-3, IL-4, IL-7, IL-23. Of 15 different chemokines examined in the study, CCL11, CCL17, CCL19, CCL20, CCL21, CCL22, CCL25, CCL26, CCL27, and CXCL9 were significantly upregulated by ORF7. These cytokines and chemokines were frequently elevated in severely ill COVID-19 patients. Our data provide an insight into how SARS-CoV-2 modulates NF-κB signaling and inflammatory cytokine expressions. The ORF7a protein may be a desirable target for strategic developments to minimize uncontrolled inflammation in COVID-19 patients.

## Introduction

Severe acute respiratory syndrome coronavirus 2 (SARS-CoV-2) is the causative agent for coronavirus disease 2019 (COVID-19) that emerged in human populations in the late December 2019. Since WHO declared the highest level of public health emergency of international concern in March 2020, more than 109 million global cases and 2.4 million deaths have been reported during the 1-year period (https://www.who.int/health-topics/coronavirus#tab=tab_1). COVID-19 involved a wide range of respiratory symptoms. Most affected people experience mild to moderate respiratory illness and recover without requiring special treatments^1^. Older people and those with underlying medical conditions like cardiovascular disease, diabetes, chronic respiratory disease, and cancer are more likely to develop serious illness due to innate and adaptive immune response disorder, tissue damages, and systemic inflammation^2,3^.

SARS-CoV-2 belongs to the *Sarbecovirus* subgenus in the *Betacoronavirus* genus*,* the *Coronavirinae* subfamily in the *Coronaviridae* family^4^. The SARS-CoV-2 genome is a single-strand positive-sense RNA of 29.9 kb in length and codes for two large polyproteins (PP1a and PP1a/b) and four structural proteins [spike (S), envelope (E), membrane (M), and nucleocapsid (N) proteins]. Two polyproteins are further processed to 16 non-structural proteins (nsp1 through nsp16) by autoproteolytic cleavages. Additionally, six potential open reading frames (ORF3a, ORF6, ORF7a, ORF7b, ORF8, and ORF10) are infused between structural genes to code for possible accessory proteins^5^. According to the data from the Global Initiative on Sharing All Influenza Data (GISAID; https://www.gisaid.org), four major clades of SARS-CoV-2 have so far been identified and named clade L (prototype virus Wuhan-Hu-1; GenBank accession number NC_045512), clade G (D614G variant of the spike protein), clade V (G251V variant of ORF3a), and clade S (L84S variant of ORF8). According to additional mutation, the clade G can be split into three subclades, clade GH (Q57H variant of ORF3a), clade GR (RG203KR variant of the nucleocapsid protein), and GV (A222V variant of the spike protein). Lastly, the rest of the sequences that are not match any of these criteria are grouped as clade O^6^.

Several studies for severe COVID-19 patients have shown the serum level elevation of some of the proinflammatory cytokines such as interleukin-1β (IL-1β), IL-6, and tumor necrosis factor (TNF)^7–11^. Such unbalanced hyperproduction of proinflammatory cytokines is linked to acute respiratory distress syndrome (ARDS) with high mortality in COVID-19 patients and often referred to as a cytokine storm^12^. ARDS is often represented by edema, gas exchange dysfunction, acute cardiac damages, respiratory failure, and secondary infection^9^. Hyperproduction of proinflammatory cytokines has been observed for other viral infections such as influenza virus H5N1, SARS-CoV-1, hantavirus pulmonary syndrome, and probably during the 1918 influenza pandemic^13–16^, and a severe outcome is resulted by a loss of negative feedback on the immune response. In turn, the cytokine secretion drives positive feedback on other immune cells and recruits more immune cells to the sites of inflammation, resulting in different organ damages. The major cytokines involved this process include interleukins, interferons, TNF, colony-stimulating factors (CSFs), the chemokine family, growth factors, and others. Evidence shows that the cytokine storm may be an important factor for disease progression, possibly leading to multiple organ failures and death, and thus, understanding the mechanism for the SARS-CoV-2-mediated hyperinflammation is an important research subject.

Proinflammatory cytokine expression is driven by the nuclear factor kappa B (NF-κB) signaling pathway^17^. NF-κB is a family of transcription factors consisting of RelA (p65), RelB, NF-κB1 (p50 and its precursor p105), NF-κB2 (p52 and its precursor p100), and c-Rel homo/heterodimers with RelA or RelB. NF-κB regulates many important cellular behaviors such as inflammatory responses, cell growth, and apoptosis. NF-κB also contributes to cancers and mitochondrial and nervous system functions. The NF-κB pathway responds to diverse stimuli including ligands of various cytokine receptors, pattern-recognition receptors (PRRs), TNF receptor (TNFR) superfamily, as well as T-cell and B-cell receptors. In turn, viruses may utilize NF-κB for their own benefits^18^. The primary mechanism for NF-κB activation is the inducible degradation of IκBα triggered by a multi-subunit IκB kinase (IKK) complex. IKK can be activated by cytokines, growth factors, mitogens, microbial components, and infectious agents. Upon stimulation, NF-κB induces a variety of proinflammatory cytokine gene expressions. These proinflammatory cytokines further activate NF-κB signaling in the autocrine manner^19^. Since proinflammatory cytokines are elevated in severe COVID-19 patients, SARS-CoV-2 seems to activate NF-κB and produces proinflammatory cytokines, which is correlated with COVID-19 pathogenesis. Indeed, NF-κB is activated in SARS-CoV-2 infected cells^20^. However, underlying mechanisms for viral modulation of NF-κB functions are still unclear.

For SARS-CoV-1, both structural proteins and accessory proteins activate NF-κB signaling. The SARS nucleocapsid (N) protein activates NF-κB and induces IL-6 expression in A549 cells^21,22^. The NF-κB response by the membrane (M) protein is controversial. In one study, the M protein suppressed the NF-κB activity in both HeLa and Vero E6 cells by affecting NF-κB nuclear translocation^23^. On the contrary, M activated the NF-κB signaling cascades and further promoted interferon-beta (IFN-β) production in HEK293T cells^24^. For accessory proteins, the ORF3a and ORF7a proteins were able to activate NF-κB and c-Jun N-terminal kinase (JNK), and significantly enhanced IL-8 expression^25^. The ORF3a protein also induced pro-IL-1β transcription through NF-κB activation and promoted NOD-like receptor (NLR)-family pyrin domain-containing 3 (NLRP3) inflammasome^26^. Given the genetic similarity of SARS-CoV-1 to SARS-CoV-2, their viral proteins may possess conserved strategies to manipulate cytokine response.

In the present study, we aimed to identify and characterize SARS-CoV-2 proteins that were able to modulate NF-κB response and inflammatory cytokine expressions. We show that the ORF3a, M, ORF7a, and N proteins of SARS-CoV-2 are NF-κB activators. No significant difference was found in NF-κB response between clade L and clade V. However, only ORF7a induced NF-κB-dictating cytokines such as IL-1α, IL-1β, IL-6, IL-8, IL-10, TNF-α, and IFNβ. The ORF7a protein also induced IL-3, IL-4, IL-7, IL-23 and 10 chemokine expressions. These cytokines and chemokines are frequently elevated in severely affected COVID-19 patients. Our results provide insight into how SARS-CoV-2 modulates NF-κB response and inflammatory cytokines expression. Our findings may be relevant to the severity of ARDS in COVID-19 patients.

## Results

### SARS-CoV-2 protein expressions in HeLa cells

**Figure 1:**
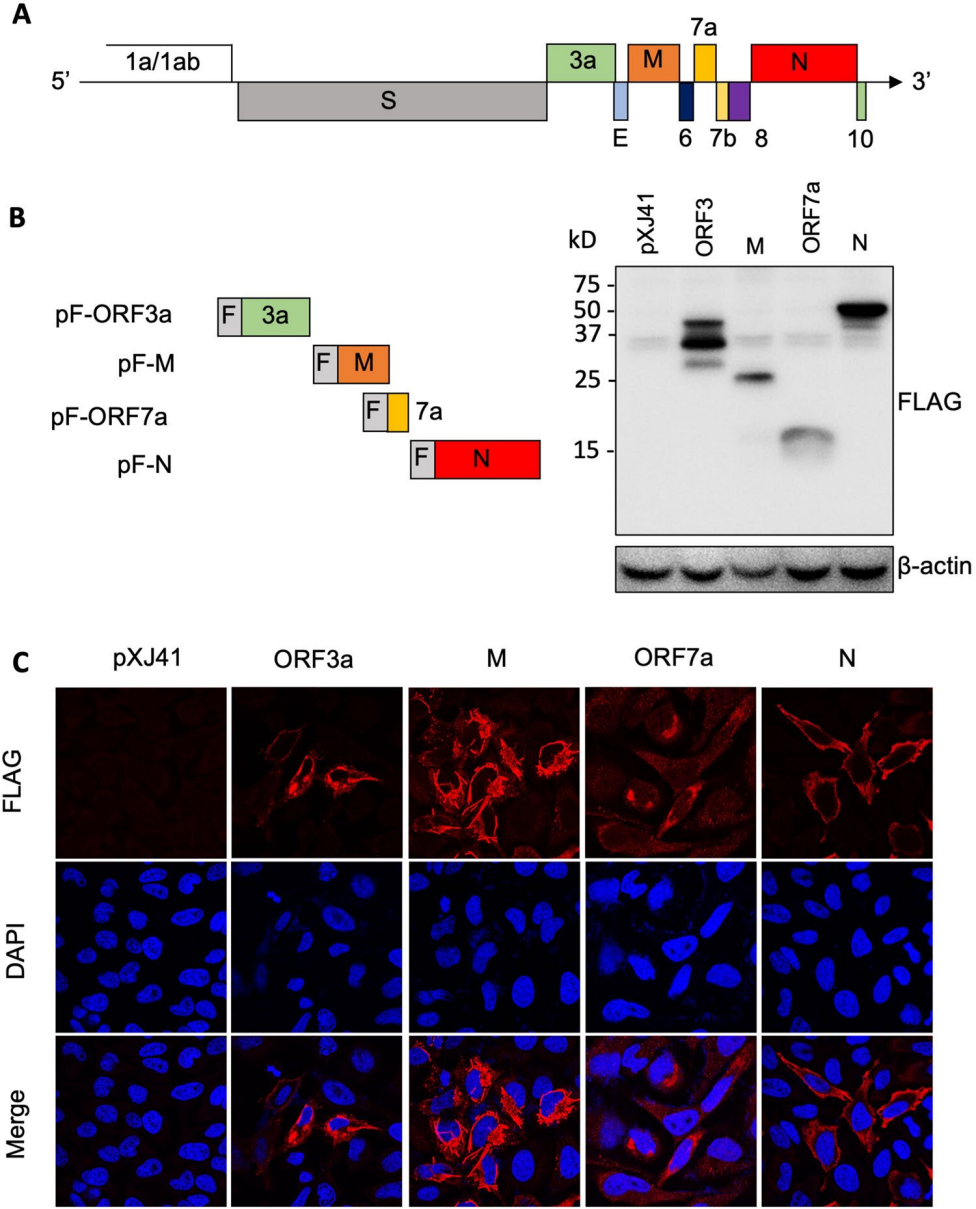
SARS-CoV-2 protein expression in HeLa cells. (**A**) Genome structure of SARS-CoV-2. The ORF1a/1ab encodes non-structure proteins, four genes encode four structural proteins (S, E, M, N), and six accessory genes encoding six accessory proteins (ORF3a, ORF6, ORF7a, ORF7b, ORF8, and ORF10) are indicated. (**B**) SARS-CoV-2 ORF3a, M, 7a, N genes were fused with a FLAG-tag (F) at the N-terminus and cloned in the pXJ41 expression vector. pF-ORF3a, pF-M, pF-ORF7a, and pF-N were transfected in HeLa cells for 24 h, and cell lysates were subjected to immunoblot using α-FLAG PAb. Beta-actin served as a loading control. The full-length blot is presented in Supplementary Fig. S1. (**C**) Distribution of SARS-CoV-2 ORF3a, M, ORF7a, and N protein in HeLa cells. Cellular localization of the individual protein (red) and cell nuclei (blue) were examined by confocal microscopy.

For SARS-CoV-1, the ORF3a, M, ORF7a, and N proteins have been reported to regulate various pathways involved in host innate immune responses^21,22,24–26^. To study SARS-CoV-2-mediated cytokine productions, we first cloned and expressed the ORF3a, M, ORF7a, and N genes from the viral RNA. Each coding sequence was fused with a FLAG-tag at the N-terminus and cloned in the pXJ41 expression vector (Fig. 1A). The constructs were individually transfected into HeLa cells or A549 cells, and their expressions were measured at 24 h post-transfection by Western blot and immunofluorescent staining using anti-FLAG antibody (Fig. 1B). Proteins of 25 K, 15 K, and 50 K in their molecular migration were identified in both cell types, and these proteins likely represented for M, ORF7a, and N, respectively. For ORF3a, three specific bands of 40 K, 35 K, and 28 K were detected by FLAG antibody, which may be due to either cryptic translation or protein cleavages. We did not pursue the nature of these bands further. The expression of each protein was confirmed by staining of cells transfected with the respective plasmid at 24 h post-transfection. The ORF3a, M, ORF7a, and N proteins were all expressed in HeLa cells and distributed in the cytoplasm (Fig. 1C).

### Activation of NF-κB by ORF3a, M, ORF7a, and N proteins of SARS-CoV-2

**Figure 2:**
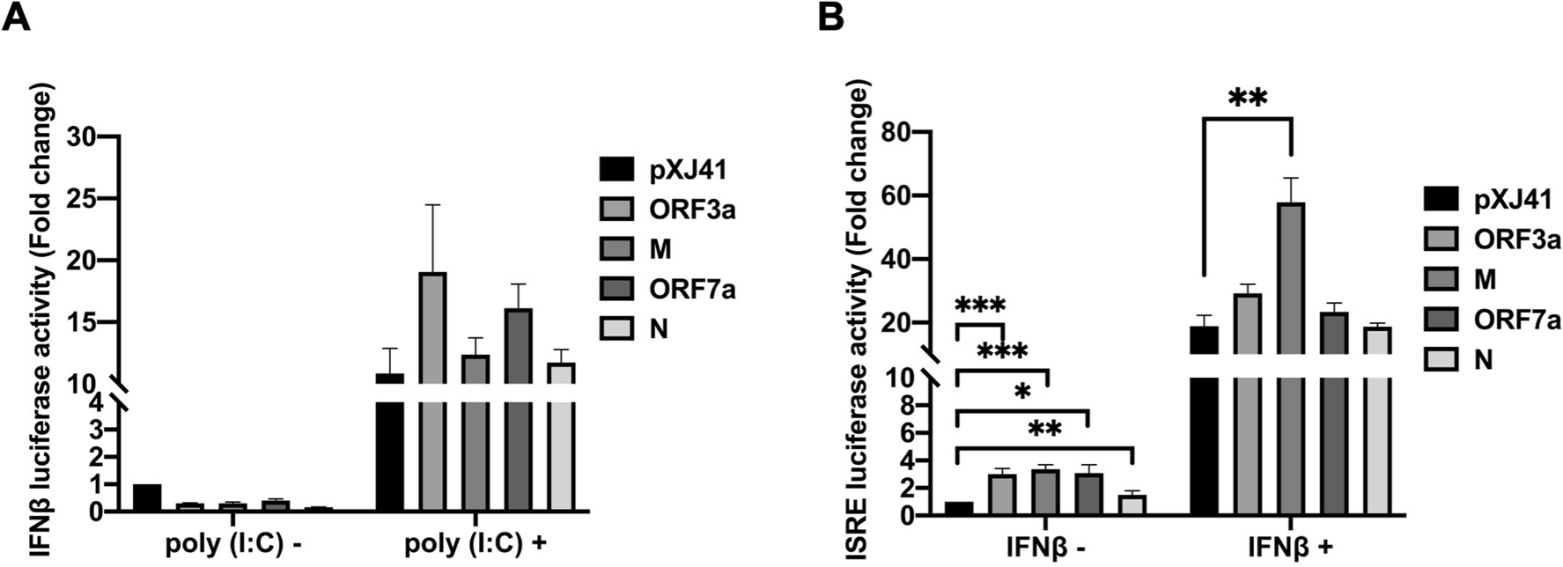
Cellular IFN response mediated by SARS-CoV-2 proteins in HeLa cells. Cells were co-transfected with pIFN-β-Luc (0.5 μg) (**A**), or pISRE-Luc (0.5 μg) (**B**), along with pRL-TK (0.05 μg) and each (0.5 μg) of indicated SARS-CoV-2 genes. At 24 h post-transfection, the cells were transfected again with 0.5 μg of poly(I:C) for stimulation for 16 h (**A**), or incubated with IFN-β (1000 UI/ml) for 6 h (**B**). Cell lysates were then prepared for luciferase assays using the Dual Luciferase assay system according to the manufacture’s instruction (Promega). Relative luciferase activities were obtained by normalizing the firefly luciferase to Renilla luciferase activities. Values of the relative luciferase activity in the pXJ41 control group were set as 1, and the values for individual viral proteins were normalized using that of the pXJ41 control. Error bars mean ± standard deviation (s.d.). (n = 3). *P < 0.05, **P < 0.01, ***P < 0.001.

The SARS-CoV-2 infection causes unbalanced inflammatory responses, characterized by weak production of type I interferons (IFN) and overexpression of proinflammatory cytokines, resulting in the ARDS and severe clinical outcome^27^. To study the role of ORF3a, M, ORF7a, and N proteins of SARS-CoV-2 for type I IFN responses, two luciferase reporter assays were employed. In the pIFN-β-Luc assay, reporter expression reflects the transcriptional activity of the IFN-β promoter and so determines the IFN production signaling. In the pISRE-Luc assay, the luciferase expression is driven by the IFN-stimulated response elements (ISRE), which is activated by type I IFNs via the JAK-STAT pathway, and thus evaluates the IFN signaling pathway^28^. HeLa cells were co-transfected with the ORF3a, M, ORF7a, or N genes and the pIFN-β-luc reporter, followed by luciferase determinations. Without stimulation with poly(I:C), a double-stranded RNA analog, the viral proteins showed no significant changes in their reporter expressions compared to pXJ41 vector control, demonstrating that none of the accessory proteins by itself is an IFN-stimulator. (Fig. 2A). Slight decreases were observed in viral protein-expressing cells without poly(I:C) stimulation and such a decrease could be due to experimental variations since the fold changes were less than 0.5. After stimulation with poly(I:C), cells expressing viral proteins showed robust IFN transcription, indicating that the ORF3a, M, ORF7a, and N proteins did not inhibit the IFN production (Fig. 2A). For the pISRE-Luc assays, ORF3a (P < 0.001, ***), M (P < 0.001, ***), ORF7a (P < 0.05, *), and N (P < 0.01, **) proteins activate ISRE without IFN stimulation (Fig. 2B). After stimulation with IFN-ß, the proteins exhibited the activation of ISRE promoter. Of these proteins, M appeared to be a more potent inducer, and the M-mediated ISRE activation was statistically significant compared to vector control (P < 0.01, **) (Fig. 2B). The two reporter assays show that the ORF3a, M, ORF7a, and N protein did not significantly activate the IFN signaling.

**Figure 3:**
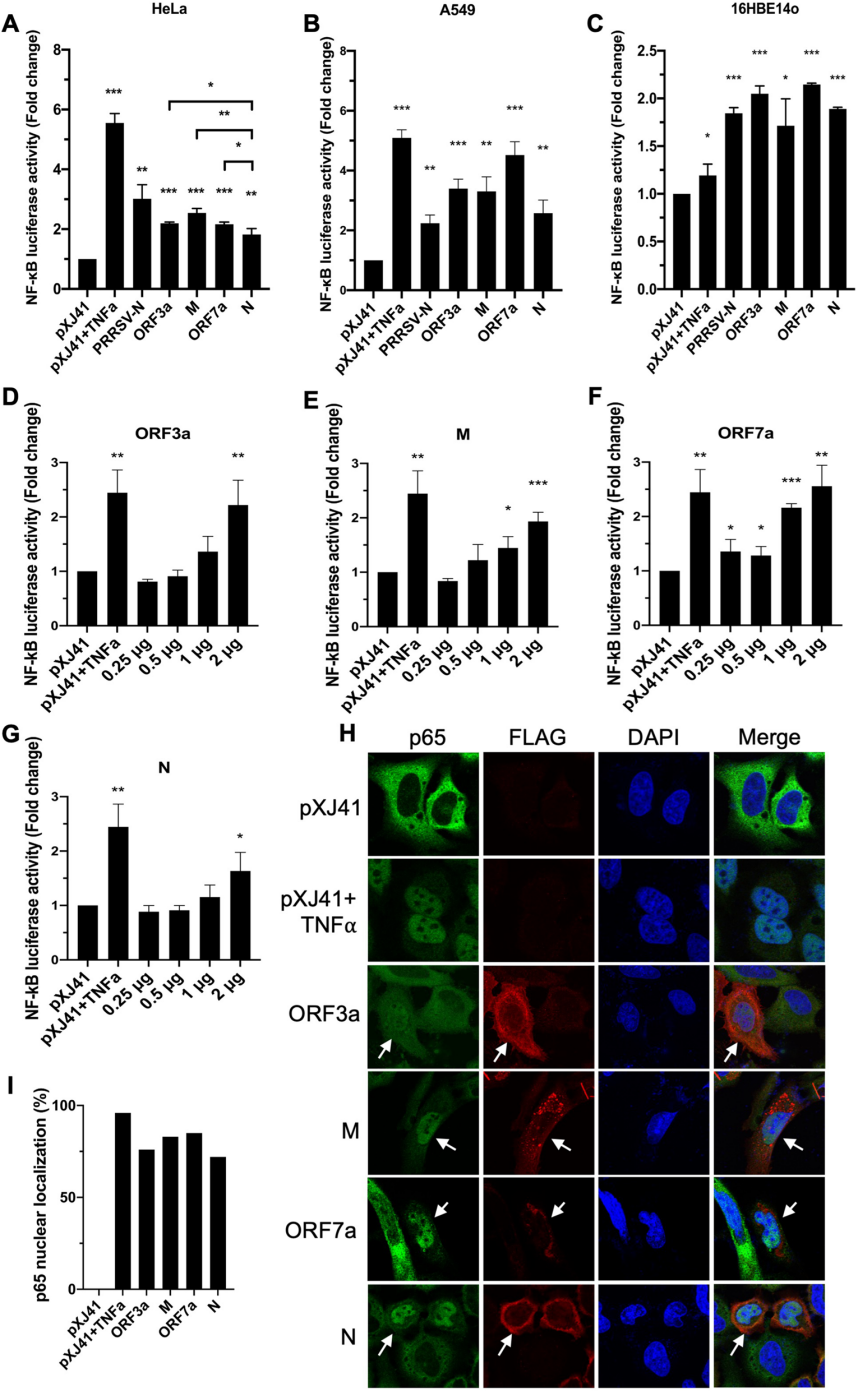
Cellular NF-κB response mediated by SARS-CoV-2 proteins in three different cell types. HeLa cells (**A,D–G**), A549 cells (**B**), or 16HBE14o cells (**C**) were co-transfected with pNF-κB-Luc (0.5 μg), pRL-TK (0.05 μg), and each of indicated SARS-CoV-2 genes. At 24 h post-transfection, cells were incubated with TNF-α (20 ng/ml) for 6 h, and cell lysates were prepared for luciferase assays. Luciferase activities were measured using the Dual Luciferase assay system according to the manufacturer’s instructions (Promega). Relative luciferase activities were obtained by normalizing the firefly luciferase to Renilla luciferase activities. Values of the relative luciferase activity in the pXJ41 control group were set as 1, and the values for individual viral proteins were normalized using that of the pXJ41 control. Error bars mean ± standard deviation (s.d.). (n = 3). *P < 0.05, **P < 0.01, ***P < 0.001. (**H**) HeLa cells were transfected with 2 μg of indicated genes for 24 h and treated or mock-treated with TNF-α (20 ng/ml) for 30 min. The cells were stained with α-p65 Mab (green) and α-FLAG PAb (red). Nuclei (blue) were stained with DAPI. White arrows indicate viral protein-expressing cells. (**I**) The percentages of cells showing p65 nuclear localization were calculated using the following formula: (Number of cells showing p65 nuclear staining)/(50 cells expressing viral protein) × 100.

To determine viral proteins that might regulate NF-κB signaling, the ORF3a, M, ORF7a, and N proteins were individually expressed and examined for their role using the NF-kB promoter-based reporter system (Fig. 3A). HeLa cells were co-transfected with the pNF-κB-luciferase reporter, pRL-TK as an internal control, and individual viral genes. The relative luciferase activities were then obtained by normalizing the firefly luciferase to *Renilla* luciferase activities. The empty vector was included as a control without TNF-α treatment, and this value set the baseline (value = 1). Nucleocapsid protein of porcine reproductive and respiratory syndrome virus (PRRSV) is known to activate NF-κB signaling, and so PRRSV N was included as the gene control. Cells were treated with TNF-α for 6 h before cell lysis, which was then used as a positive control. Compared to the vector control, the TNF-α treatment stimulated the reporter activity by 5.5-folds, indicating the normal activation of NF-κB signaling in the assay system. For SARS-CoV-2 viral proteins, each of the ORF3a, M, and ORF7a proteins induced NF-κB activation significantly by more than two-fold (2.196-, 2.542-, 2.161-fold, respectively) (Fig. 3A). The N protein also activated NF-κB by nearly two-fold (1.823-fold). Although those values were not as high as that of TNF-α stimulation, they were statistically significant (P < 0.01, **), and the results indicated the ORF3a, M, ORF7a, and N proteins were NF-κB activators, being ORF3a, M, and ORF7a more potent than N (ORF3a vs N and ORF7a vs N, P < 0.05; M vs N, P < 0.01). The NF-κB activation were then determined using varying amounts of viral proteins. The activations were positively correlated with the increasing amounts of proteins (Figs. 3D–G), indicating the dose-dependent activation of NF-κB by SARS-CoV-2 ORF3a, M, ORF7a, and N.

Since SARS-CoV-2 does not naturally infect HeLa cells^29^, the NF-κB activation was further examined in two other cell types. A549 cells are human alveolar basal epithelial cells and are naturally non-permissive for SARS-CoV-2. However, they become permissive for infection when angiotensin-converting enzyme 2 (ACE2), which is the receptor for SARS-CoV-2, is overexpressed^30^. A549 cells were co-transfected with the pNF-κB-luciferase reporter, pRL-TK internal control, and individual viral genes for 24 h, and the reporter expression was examined. ORF3a, M, ORF7a, and N significantly induced NF-κB activation by more than two-fold in A549 cells compared to the control group (Fig. 3B). We next examined the NF-kB activation in 16HBE14o cells. These cells are human bronchial epithelial cells and are naturally permissive for SARS-CoV-2^31^. Compared to HeLa and A549 cells, TNF-α treatment did not significantly stimulate the luciferase activity in 16HBE14o cells. However, the SARS-CoV-2 ORF3a, M, ORF7a, and N proteins significantly induced NF-κB activation by more than 1.5-fold compared to the control group in 16HBE14o cells (Fig. 3C), indicating that these viral proteins activate the NF-kB signaling regardless of different cell types.

This finding was confirmed by examining the p65 nuclear localization. NF-κB activation is mediated by p65 (RelA) translocation to the nucleus^17^. Thus, it was of interest to confirm the NF-κB activation by examining the subcellular distribution of p65. The p65 protein was stained with anti-p65 PAb (Fig. 3E, in green), and the viral proteins were stained with anti-FLAG Ab (in red). Similar to the evidence that endogenous p65 was localized in the nucleus when stimulated with TNF-α, cells expressing individual SARS-CoV-2 proteins distributed p65 predominately to the nucleus despite the absence of TNF-α stimulation (Fig. 3E, arrows), indicating the activation of p65 by ORF3a, M, ORF7a, and N proteins of SARS-CoV-2. The percentages of p65 nuclear transport-positive cells were calculated, and 76%, 83%, 85%, and 72% of cells showed p65 nuclear translocation for ORF3a, M, ORF7a, and N, respectively (Fig. 3I). Taken together, these results demonstrate that the four proteins can promote NF-κB activation.

### NF-κB-mediated proinflammatory cytokine productions by SARS-CoV-2 proteins

**Figure 4:**
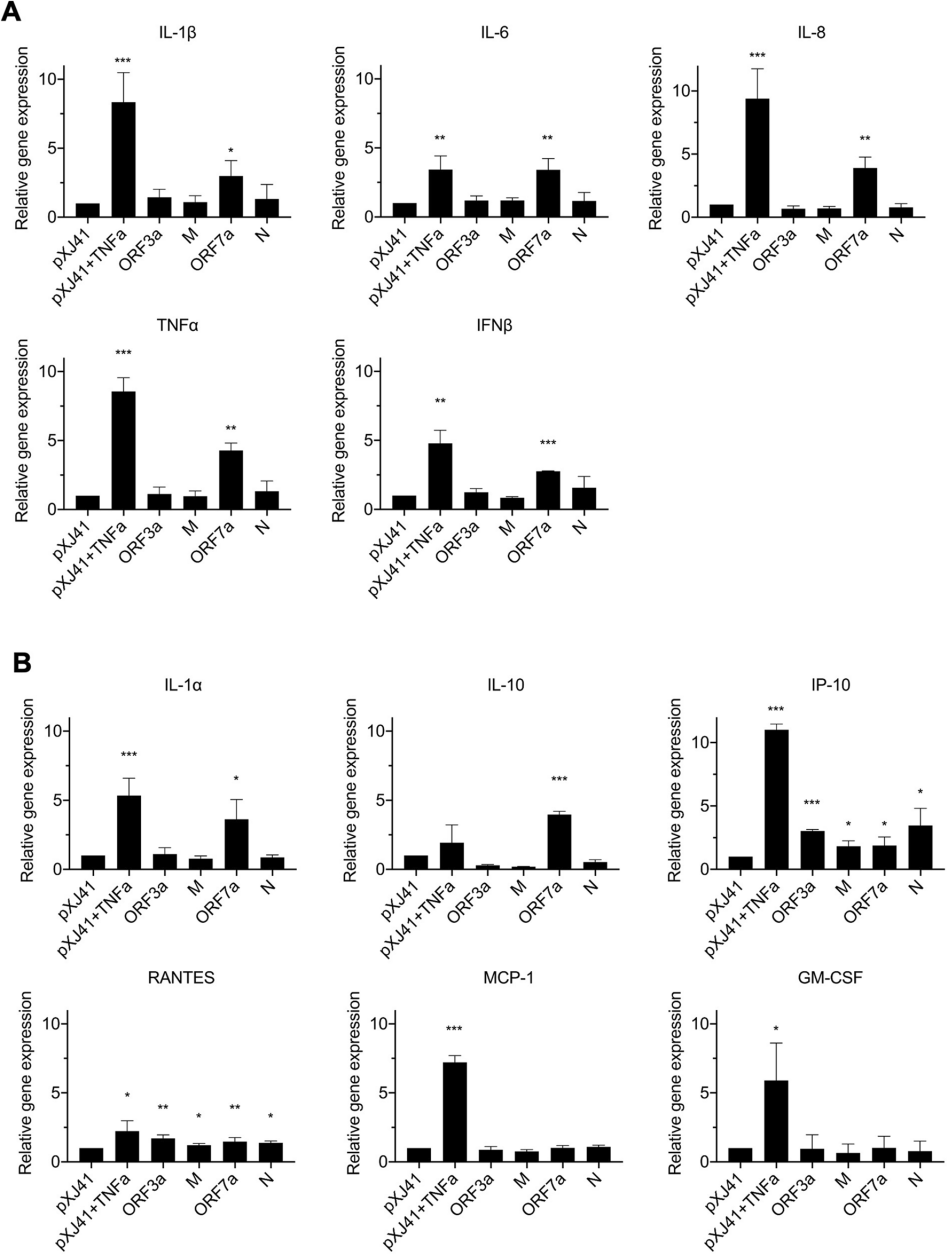
NF-κB related cytokine response. HeLa cells were transfected with 2 μg of indicated genes for 24 h and treated with or mock-treated with TNF-α (20 ng/ml) for 6 h. The expression levels of proinflammatory cytokines (**A**) and inflammatory cytokines (**B**) were calculated with 2^−ΔΔCT^ method by normalizing to that of GAPDH. The fold changes were calculated with respect to the level of pXJ41. Error bars mean ± s.d. (n = 3). *P < 0.05, **P < 0.01, ***P < 0.001.

Unbalanced hyperproduction of proinflammatory cytokines has been observed in COVID-19 patients^7–11^. One of the NF-κB functions is the regulation of some of the proinflammatory cytokine expressions, and thus, we examined NF-κB-mediated proinflammatory cytokine gene expression. Cells were transfected with individual viral genes for 24 h, and specific transcripts were quantitated by RT-qPCR (Fig. 4). When proinflammatory cytokines were examined (Fig. 4A), the ORF7a protein significantly upregulated the IL-1β (P < 0.05, *), IL-6 (P < 0.01, **), IL-8 (P < 0.01, **), TNF-α (P < 0.01, **), and IFN-β (P < 0.001, ***) transcriptions. It was interesting to note that the ORF3a, M, and N proteins did not activate those cytokines. These data demonstrate that the ORF7a protein activates the NF-κB signaling and promotes major proinflammatory cytokine productions.

We also determined the expression of other cytokines produced via NF-κB signaling (Fig. 4B). The results showed that ORF7a stimulated IL-1α and IL-10 transcriptions, and their increases were statistically significant (P < 0.05 and P < 0.001, respectively). For IP-10 and RANTES, the statistical analysis showed that the ORF3a, M, ORF7a, and N proteins induced significant levels of expression compared to those of vector control (Fig. 4B). However, the fold changes were below 1.5 to 2.0, and we concluded that upregulations of IP-10 and RANTES by these viral proteins were insignificant. These viral proteins did not induce MCP-1 and GM-CSF expressions (Fig. 4B). Taken together, our data conclude that the ORF7a protein of SARS-CoV-2 is the potent activator for the NF-κB-mediated inflammatory cytokine productions.

### Other cytokine and chemokine responses mediated by ORF7a protein

**Figure 5:**
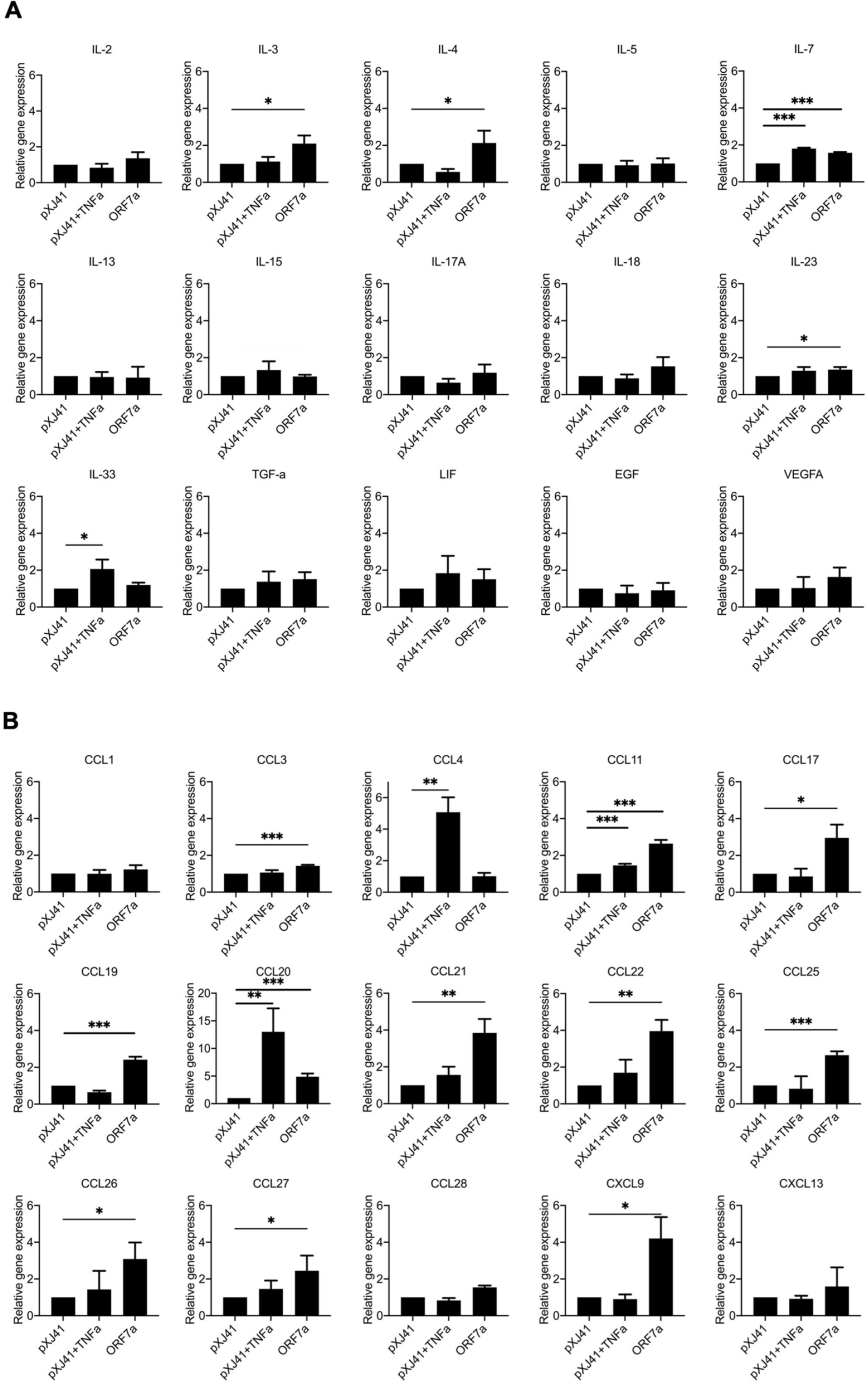
Inflammatory cytokine responses to SARS-CoV-2 ORF7a protein. HeLa cells were transfected with 2 μg of indicated genes for 24 h and treated with or mock-treated with TNF-α (20 ng/ml) for 6 h. The expression levels of (**A**) cytokines and (**B**) chemokines were calculated with 2^−ΔΔCT^ method by normalizing to that of GAPDH. The fold changes were calculated with respect to the level of pXJ41. Error bars mean ± s.d. (n = 3). *P < 0.05, **P < 0.01, ***P < 0.001.

Since the ORF7a protein appeared to be the most potent inflammatory cytokine activator (Fig. 4), we expanded the ORF7a-mediated regulation to 30 additional cytokines and chemokines. These cytokines are elevated in COVID-19 patients, but it is unknown which viral proteins are responsible for the elevation^10,20^. Of 11 different interleukins, IL-3, IL-4, IL-7, and IL-23 showed significant upregulation by the ORF7a protein compared to vector control (Fig. 5A). Of 15 various chemokines, CCL11, CCL17, CCL19, CCL20, CCL21, CCL22, CCL25, CCL26, CCL27, and CXCL9 were significantly upregulated by ORF7 (Fig. 5B). These results demonstrate that ORF7a protein mediates different cytokine and chemokine activations, partially representing the cytokine chemokine profiles in COVID-19 patients during infection.

### NF-κB activation by ORF3a from clade V of SARS-CoV-2

**Figure 6:**
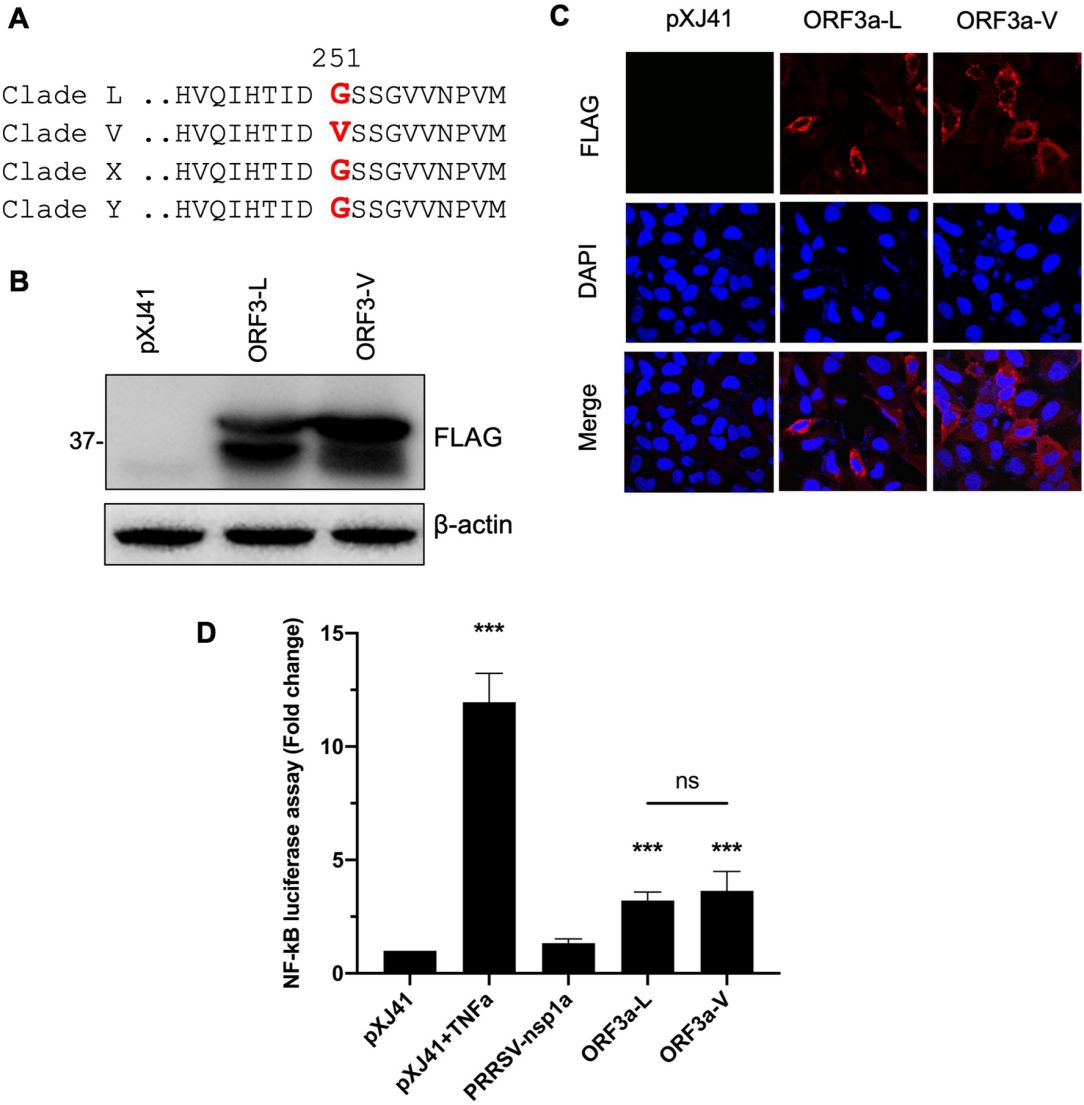
NF-κB activation by ORF3a from different clades of SARS-CoV-2. (**A**) Sequence alignments of four major clades of SARS-CoV-2 ORF3a. Single amino acid change (G251V) was identified in clade V. ORF3a genes from clade L and V were fused with the FLAG-tag and cloned in the pXJ41 expression vector and designated as ORF3a-L and ORF3a-V. Using α-FLAG PAb, expression patterns of ORF3a-L and ORF3a-V were demonstrated by immunoblot (**B**) or IFA (**C**). Beta-actin served as a loading control. The full-length blot of (**B**) is presented in Supplementary Fig. S2. (**D**) Cells were co-transfected with pNF-κB-Luciferase (0.5 μg), pRL-TK (0.05 μg), and each (0.5 μg) of indicated SARS-CoV-2 ORF3a genes for 24 h. Cells were treated or mock-treated with TNF-α (20 ng/ml) for 6 h, and cell lysates were used for luciferase assays. Relative luciferase activities were obtained by normalizing the firefly luciferase to Renilla luciferase activities. Values of the relative luciferase activity in the pXJ41 control group were set as 1, and the values for individual viral proteins were normalized using that of the pXJ41 control. Error bars mean ± standard deviation (s.d.). (n = 3). *ns* non-significance (P > 0.05), ***P < 0.001.

Genetic variants were described for SARS-CoV-2, and those variants were grouped into different clades. Furthermore, SARS-CoV-2 has been shown to infect various animal species in addition to humans, implicating evolutionary mutations and potential adaptation in animal hosts^32^. In April 2020, five tigers and three lions at the Bronx zoo in New York City tested positive for COVID-19^33^. By sequencing, the viruses infecting tigers and lions were found to belong to different clades^33^. ORF3a sequences from affected tigers were identical to that of Wuhan-Hu-1 which is clade L. ORF3a sequences from the affected lions belonged to clade V as defined by the G251V substitution^33^. Thus, ORF3 sequences of clade V differed from that of clade L which was used in the present study. Since the G251 variant of ORF3a is unique in clade V (Fig. 6A), it was of interest to see if a single amino acid change from G to V at position 251 of ORF3a would affect the NF-κB activation. The ORF3a gene was cloned from an affected lion and inserted into pXJ41 with a FLAG tag at the N-terminus. The ORF3a gene from clade L was designated ORF3-L, and the gene from clade V was named ORF3-V. Both constructs were transfected to HeLa cells, and their protein expressions were confirmed by Western blot (Fig. 6B) and IFA (Fig. 6C) using anti-FLAG tag antibody. The ORF3a proteins from ORF3-L and ORF3-V were then individually examined for NF-kB activation in the NF-κB-Luc assay (Fig. 6D). Both clades of ORF3a induced NF-κB activation significantly by more than two-fold, but no significant difference was observed between ORF3-L and ORF3-V. We concluded that G251V mutation in ORF3a did not change the property for NF-κB activation. This finding implicates that NF-κB function may be maintained for SARS-CoV-2 and is conserved among genetic variants.

## Discussion

Proinflammatory cytokines play a vital role in the host immune response against infecting pathogens, but unbalanced hyperproduction of these cytokines may cause tissue damages and severe clinical outcomes. Numerous clinical studies have described abnormally high levels of cytokines in the COVID-19 patients. These cytokines include IL-1, IL-6, IL-8, TNF-α, and other cytokines and chemokines^9,10,20,34^. NF-κB dictates the expression of proinflammatory cytokines, and they further activate NF-κB by a positive feedback mechanism^17^. In COVID-19 patients, highly expressed proinflammatory cytokines further stimulates NF-kB^20^. The NF-κB upregulation probably contributes to inflammatory response, as seen in COVID-19 patients. The viral proteins activating this response and the molecular mechanisms for such activation remained unknown, and in the present study, we identified four SARS-CoV-2 proteins as the NF-κB activators. Of these four, ORF7a was the most potent NF-kB inducer and thus proinflammatory cytokine producer.

Compared to other respiratory viruses, SARS-CoV-2 is a poor inducer of type I IFNs in vitro and in animal models^30,35^. Recent reports show that some of the SARS-CoV-2 proteins can antagonize IFN response by distinct mechanisms, and nsp1, nsp6, nsp13-15, and ORF6 have been identified as viral IFN antagonists^36,37^. In the present study, we have shown that the ORF3a, M, ORF7a, and N proteins of SARS-CoV-2 do not directly inhibit nor activate the IFN response (Fig. 2A). However, ORF7a protein induced the production of IFNβ mRNA (Fig. 4A), indicating that ORF7a mediates the NF-κB pathway and may stimulate the IFNβ response instead of direct induction of IFNβ. Xia et al. (2020) report that ORF3a, M, ORF7a suppressed IFN signaling^36^. However, in our studies, the ORF3a, M, ORF7a, and N proteins did not suppress the IFN signaling in HeLa cells (Fig. 2B). The M protein even induced the ISRE response after IFN stimulation, implicating the absence of suppression of JAK-STAT signaling. SARS-CoV-2 increased the high-level production of proinflammatory cytokines in COVID-19 patients^9,10,20,34^. The studies for SARS-CoV-1 ORF3a, M, ORF7a, and N proteins have shown upregulation of NF-κB activity^21,22,24–26^. In the present study, we have identified using the reporter assay that ORF3a, M, ORF7a, and N of SARS-CoV-2 were NF-κB activators and confirmed the findings by showing the p65 nuclear localization in the respective gene expressing cells (Fig. 3). From these data, it is evident that NF-κB is upregulated by the ORF3, M, ORF7a, and N proteins.

In many COVID-19 patients, levels of cytokines and chemokines are elevated^7–11^. IL-1β, IL-6, IL-8, and TNF are proinflammatory cytokines dictated by NF-κB, and they are hyper produced. These proinflammatory cytokines are linked to cytokine release syndrome (CRS), implicating the positive associations with severe disease outcome^10^. Our study shows that ORF7a of SARS-CoV-2 can activate NF-κB function and increases proinflammatory cytokine expressions (Fig. 4). Since IL-1β is in part responsible for the cytokine storm by SARS-CoV-1 and MERS-CoV^38,39^, ORF7a may play a significant role in the clinical severity of COVID-19. Besides NF-κB-mediated inflammatory cytokines, ORF7a also induces a variety of interleukins and chemokines (Fig. 5). The increase of cytokines and chemokines was also reported for COVID-19 patients^10^, which is supportive and consistent with our findings.

Furthermore, the patients who ultimately died of COVID-19 exhibited significantly elevated levels of chemokines associated with monocytes and T cell recruitments and survival, such as CXCL9 and CCL21^10^. Indeed, we have shown that the ORF7a protein elevates CXCL9 and CCL21 expressions significantly (Fig. 5). These results indicate that the ORF7a protein is an essential viral factor that manipulates cytokine and chemokine responses during SARS-CoV-2 infection. The molecular basis for activation of the cytokines by ORF7a warrants further studies.

In summary, we have identified the NF-κB activators of SARS-CoV-2. The ORF3a, M, ORF7a, N proteins can upregulate NF-κB activity, and ORF7a is the most potent activator for inflammatory cytokines expressions. Our finding provides insight into the molecular basis of cytokine storms in COVID-19 patients. The ORF7a protein may be a desirable target to develop a strategy to minimize uncontrolled inflammation in COVID-19 patients.

## Materials and methods

### Cells, antibodies, and chemicals

HeLa (NIH AIDS Research and Reference Reagent Program, Germantown, MD) cells were maintained in Dulbecco’s modified Eagle’s medium (DMEM; Corning Inc., Corning, NY), supplemented with 10% heat-inactivated fetal bovine serum (FBS; Gibco, Grand Island, NY), in a humidified incubator with 5% CO2 at 37 °C. A549 cells and 16HBE14o cells (Kindly provided by Gee Lau, University of Illinois) were maintained in Minimum Essential Medium (MEM; Corning Inc., Corning, NY), supplemented with 10% heat-inactivated FBS, in a humidified incubator with 5% CO2 at 37 °C. Anti (α)-FLAG PAb (rabbit) was purchased from Rockland Inc (Gilbertsville, PA). α-p65 Mab (mouse) (F-6, sc-8008) was purchased from Santa Cruz Biotechnologies Inc. (Santa Cruz, CA). Alexa-Flour 488-conjugated, and Alexa-Flour 568-conjugated secondary antibodies were obtained from ThermoFisher (Rockford, IL). Human tumor necrosis factor-α (TNF-α) (8902) was purchased from Cell Signaling (Danvers, MA). DAPI (4′, 6′-diamidino-2-phenylindol) was obtained from Sigma (St. Louis, MO).

### Genes and plasmids

The pUC57 plasmids, which contained each of SARS-CoV-2 viral protein coding sequences, were obtained from Sangon Biotech (Shanghai, China). The SARS-CoV-2 virus RNA isolated from infected lion feces was obtained from the Veterinary Diagnostic Laboratory, (University of Illinois, Urbana, IL)^33^. The coding sequences for ORF3, ORF7, M, E. N of SARS-CoV-2 were amplified by PCR using primer pairs containing the ATG translation initiation and TAG termination codons. A FLAG-tag (5′-GATTACAAGGATGACGACGATAAG-3′) was added in the forward primer such that the tag would be included at the N-terminus of each gene. The coding sequences were cloned in the mammalian expression vector pXJ41 using the Eco RI- and Xho I-recognition sequences or Not I- and Bam HI-recognition sequences. The reporter plasmids pIFN-β-Luc was kindly provided by Stephan Ludwig (Institute of Molecular Medicine, Heinrich Heine Universtät, Düsseldorf, Germany)^40^. The pISRE-Luc and pNF-κB-Luc was obtained from Stratagene Inc (La Jolla, CA). The pRL-TK Renilla luciferase reporter plasmid was purchased from Promega (Madison, WI).

### DNA transfection and dual luciferase assay

DNA transfection was performed using Lipofectamine 200 according to the manufacturer’s instruction (Invitrogen). Cells were seeded in 12-well plates. In each well, 0.5 μg of pIFN-β-Luc, or pISRE-Luc, or pNF-κB-Luc, 0.05 μg of pRL-TK, and 0.5 μg of the gene of interest were co-transfected. For IFN-β luciferase assay, 0.5 μg of poly(I:C) was transfected into cells for stimulation for 16 h at 24 h after DNA transfection. For ISRE luciferase assay or NF-κB luciferase assay, at 24 h post-transfection, cells were stimulated with 1000 UI/ml of IFN-β or 20 ng/ml of TNF-α for 6 h, and lysates were prepared using Passive lysis buffer (Promega). Supernatants were collected and measured for luciferase activities using the Dual luciferase reporter assay system (Promega). Signals were determined in the luminometer (Wallac 1420 VICTOR multi-label counter, Perkin Elmer, Waltham, MA). Values for firefly luciferase reporter activities were normalized by the Renilla internal control, and results were expressed as relative luciferase activities. The assay was repeated twice, and each assay was conducted in triplicate.

### Immunofluorescence assay (IFA)

HeLa cells were grown on coverslips for 16 h. Cells were transfected with 2 μg of plasmid DNA for 24 h. For p65 nuclear translocation, cells were stimulated by incubation with 20 ng/ml of TNF-α for 30 min at 24 post-transfections. Cells were fixed with 4% paraformaldehyde in phosphate buffered saline (PBS) at room temperature (RT). Following washing with PBS, cells were permeabilization for 10 min using 0.1% Triton X-100 in PBS at RT. After blocking with 1% bovine serum albumin (BSA) in PBS for 1 h at RT, cells were incubated with the primary antibodies in blocking buffer for 2 h at RT, followed by three washes with PBS and incubation with the secondary antibodies for 1 h at RT. Nuclei were stained with DAPI in 1:5000 dilution for 10 min. After staining, coverslips were mounted on microscope slides in the Fluoromount-G mounting medium (Southern Biotech, Birmingham, AL). Stained cells were examined under the laser scanning confocal microscope (Nikon A1R).

### Immunoblotting

Cell lysates were prepared in the RIPA buffer (50 mM Tris/HCl [pH 8], 150 mM NaCl, 1% NP-40, 1% SDS, 0.5% sodium deoxycholate) containing 1× protease inhibitor cocktail (Promega) and centrifuged at 4 °C for 10 min at 12,000 rpm. Cell lysates were resolved by 10% SDS-PAGE followed by transfer to Immobilon-P PVDF membrane (Millipore, Temecula, CA). After incubation with TBST blocking buffer (10 mM Tris–HCl, 150 mM NaCl, 0.05% Tween-20 containing with 5% skim milk powder) for 1 h at RT, membranes were incubated with primary antibody at 4 °C overnight. The membranes were washed five times with TBST and incubated with peroxidase-conjugated secondary antibody in TBST for another 1 h at RT. After five washes with TBST, proteins were visualized using the ECL detection system (Thermo, Minneapolis, MN).

### Reverse transcription-quantitative PCR (RT-qPCR)

Total cellular RNA was extracted using the TRIzol reagent according to the manufacturer’s instruction (Invitrogen). RT-qPCR was performed in the ABI sequence Detector System (ABI Prism 7000 Sequence Detection System and software: Applied Biosystems) using a final volume of 25 μl containing 2 μl of cDNA from reverse-transcription reaction, a primer mix (2.5 pM each of sense and antisense primers), 12.5 μl of SYBR Green Master Mix (Applied Biosystems), and 8 μl of distilled water. The primer sequences were listed in Supplementary Table S1. The amplification parameters were 40 cycles of two steps each cycle comprised of heating to 95 °C and 60 °C. The mRNA levels were calculated using the 2^−ΔΔCT^ method^41^ and normalized using GAPDH.

### Statistical analysis

Statistical significance was determined by two-tailed Student’s t-test, and analyses were performed using GraphPad Prism version 8.00 (San Diego California USA).

## Supplementary Information

Supplementary Information.
