# Supplementary material for: Activation of NF-κB and induction of proinflammatory cytokine expressions mediated by ORF7a protein of SARS-CoV-2
Source: Sci Rep. 2021 Jun 29;11:13464. doi: 10.1038/s41598-021-92941-2 (PMC8242070; doi:10.1038/s41598-021-92941-2)

Activation of NF-kB and Induction of Proinflammatory Cytokine Expressions Mediated by ORF7a Protein of SARS-CoV-2

Chia-Ming Su[1)], Leyi Wang[2)], Dongwan Yoo[1), *]

[1)] Department of Pathobiology, [2)] Veterinary Diagnostic Laboratory and Department of Veterinary Clinical Medicine, University of Illinois at Urbana-Champaign, Urbana, IL USA

*Corresponding author mailing address:

Dongwan Yoo

Department of Pathobiology

University of Illinois at Urbana-Champaign

2001 Lincoln Ave, Urbana, IL 61802 USA

Email: dyoo@illinoois.edu

Phone: 217-244-9120

Supplementary Table S1. Primer sequences for RT-qPCR

Primer name	Primer sequence
hIL-6-F	5'-GTAGCCGCCCCACACAGA-3'
hIL-6-R	5'-CATGTCTCCTTTCTCAGGGCTG-3'
hIL-8-F	5'-ATAAAGACATACTCCAAACCTTTCCAC-3'
hIL-8-R	5'-AAGCTTTACAATAATTTCTGTGTTGGC-3'
hTNF-α-F	5'-TCCTCAGCCTCTTCTCCTTCCT-3'
hTNF-α-R	5'-ACTCCAAAGTGCAGCAGACAGA-3'
hIL-1b-F	5'-AAATACCTGTGGCCTTGGGC-3'
hIL-1b-R	5'-TTTGGGATCTACACTCTCCAGCT-3'
hRANTES-F	5'-TTTGCCTACATTGCCCGC-3'
hRANTES-R	5'-TTTCGGGTGACAAAGACGACT-3'
hGM-CSF-F	5'-AGACACTGCTGCTGAGATGAA-3'
hGM-CSF-R	5'-AGGCCCTGCTTGTACAGCT-3'
hIL-1a-F	5'-ATCAGTACCTCACGGCTGCT-3'
hIL-1a-R	5'-TGGGTATCTCAGGCATCTCC-3'
hIFN-b-F	5'-GATTCATCTAGCACTGGCTGG-3'
hIFN-b-R	5'-CTTCAGGTAATGCAGAATCC-3'
hIP-10-F	5'-TGACTCTAAGTGGCATTCAAGGAG-3'
hIP-10-R	5'-TTTTTCTAAAGACCTTGGATTAACAGG-3'
hMCP1-F	5'-ACTCTCGCCTCCAGCATGAA-3'
hMCP1-R	5'-TTGATTGCATCTGGCTGAGC-3'
hIL-10-F	5'-TCATCGATTTCTTCCCTGTG-3'
hIL-10-R	5'-ATGGCTTTGTAGATGCCTTT-3'
IL-2-F	5'-GAATCCCAAACTCACCAGGATGCTC-3'
IL-2-R	5'-TAGCACTTCCTCCAGAGGTTTGAGT-3'
IL-3-F	5'-GCGATCTTTTGAGTCCAACG-3'
IL-3-R	5'-GCTTCTGGTCCTGGAATGTG-3'
IL-4-F	5'-CCAACTGCTTCCCCCTCTG-3'
IL-4-R	5'-TCTGTTACGGTCAACTCGGTG-3'
IL-5-F	5'-AAGAGACCTTGGCACTGCTTTC-3'
IL-5-R	5'-GGAACAGGAATCCTCAGAGTCTCA-3'
IL-7-F	5'-TTGGACTTCCTCCCCTGATCC-3'
IL-7-R	5'-TCGATGCTGACCATTAGAACAC-3'
IL-13-F	5'-TGAGGAGCTGGTCAACATCA-3'
IL-13-R	5'-CAGGTTGATGCTCCATACCAT-3'
IL-15-F	5'-TTTCAGTGCAGGGCTTCCTAA-3'
IL-15-R	5'-GGGTGAACATCACTTTCCGTAT-3'

IL17A-F	5’-CAATCCCACGAAATCCAGGATG-3’
IL17A-R	5’-GGTGGAGATTCCAAGGTGAGG-3’
IL18-F	5’-GATAGCCAGCCTAGAGGTATGG-3’
IL18-R	5’-AAGAAGTGCTCGTCCTCGTC-3’
IL-23-F	5’-GGACAACAGTCAGTTCTGCTT-3’
IL-23-R	5’-CACAGGGCTATCAGGGAGC-3’
IL-33-F	5’-GTTACTTTAGGAGAGAAACCACCAAA-3’
IL-33-R	5’-TTGACAGGCAGCGAGTACCA-3’
CXCL9-F	5’-TTGCTGGTTCTGATTGGAGTG-3’
CXCL9-R	5’-AAGGTCTTTCAAGGATTGTAGGTG-3’
CXCL13-F	5’-TTGAGGTGTAGATGTGTCCAAGA-3’
CXCL13-R	5’-ATTCGATCAATGAAGCGTCTAGG-3’
CCL1-F	5’-TGGATGGGTTCAGAGGCACA-3’
CCL1-R	5’-AGGGCAGAAGGAATGGTGTAG-3’
CCL3-F	5’-TCTGGTGACAACCGAGTGGC-3’
CCL3-R	5’-CCGATCACAGCCCTGAACAA-3’
CCL4-F	5’-CCTCGCAACTTTGTGGTAGA-3’
CCL4-R	5’-CAGTTCAGTTCCAGGTCATACAC-3’
CCL11-F	5’-ATACCCCTTCAGCGACTAGAG-3’
CCL11-R	5’-GCTTTGGAGTTGGAGATTTTTGG-3’
CCL17-F	5’-CCAGGGATGCCATCGTTT-3’
CCL17-R	5’-GGTGGAGGTCCCAGGTAGTC-3’
CCL19-F	5’-AAGACTGCTGCCTGTCTGTGA-3’
CCL19-R	5’-CTGGATGATGCGTTCTACCC-3’
CCL20-F	5’-GACATAGCCCAAGAACAGAAA-3’
CCL20-R	5’-GACAAGTCCAGTGAGGCACAA-3’
CCL21-F	5’-TGAAGCCTGAACCCAAGATG-3’
CCL21-R	5’-CAGCCATGCAGGGTAGAGC-3’
CCL22-F	5’-GGAGGCAAAGAGTAGGGTGTAAT-3’
CCL22-R	5’-TCAGCCAGAAAGGCATAGATAGA-3’
CCL25-F	5’-CTGGCAACTGGAAAGAGGGAG-3’
CCL25-R	5’-GGACGGCAAGAAACAGAAAGTG-3’
CCL26-F	5’-ATCAGGCCCTTCTCAGGTTT-3’
CCL26-R	5’-AATTGAGGCTGAGCCAAAGA-3’
CCL27-F	5’-AGGCTGAGCAACATGAAGGG-3’
CCL27-R	5’-TGCTGGGTGGCAGTAGGAAT-3’
CCL28-F	5’-CATACTTCCCATTGCCTCCA-3’
CCL28-R	5’-TGCCCTGTTACTGTTCCTCTT-3’
TGF-α-F	5’-GAGCCCTCGGTAAGTATGTTTAG-3’

TGF-α-R	5’-CATAGTGGAGGTGACTTGTTAGAG-3’
LIF-F	5’-CCCATCACCCCTGTCAACG-3’
LIF-R	5’-GGGCCACATAGCTTGTCCA-3’
EGF-F	5’-AAGGTACTCTCGCAGGAAATGG-3’
EGF-R	5’-ACATACTCTCTCTTGCCTTGACC-3’
VEGFA-F	5’-ATCACGAAGTGGTGAAGTTC-3’
VEGFA-R	5’-TGCTGTAGGAAGCTCATCTC-3’
hGAPDH-F	5’-CGGAGTCAACGGATTTGGTCGTA-3’
hGAPDH-R	5’-AGCCTTCTCCATGGTGGTGAAGAC-3’

Supplementary Figure S1. SARS-CoV-2 protein expression.

The ORF3a, M, 7a, N genes of SARS-CoV-2 were fused with a FLAG-tag at the N-terminus and transfected into HeLa cells for 24 h. Cell lysates were prepared and subjected to immunoblot using α-FLAG PAb. This figure presents the full-length gel and image of the blots for Fig. 1b.

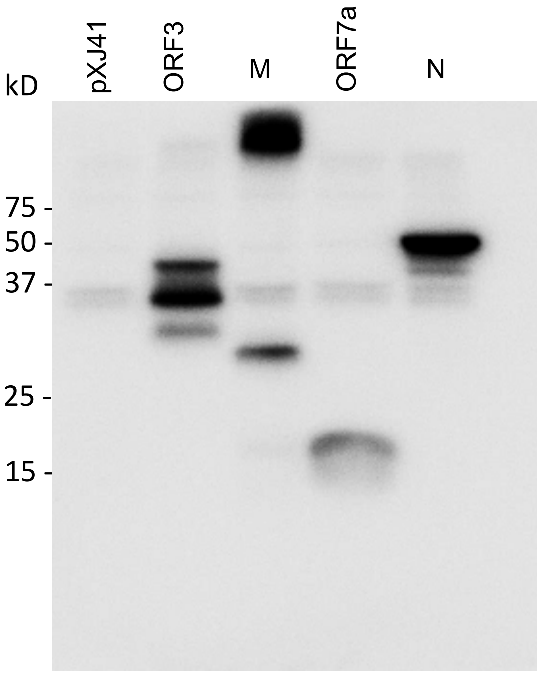

Supplementary Figure S2. ORF3a protein expressions of clades L and V of SARS-CoV-2. ORF3a genes from clades L and V were fused with a FLAG-tag, cloned in the pXJ41 expression vector, and designated as ORF3a-L and ORF3a-V, respectively. ORF3a-L and ORF3a-V genes were transfected into HeLa cells for 24 h, and cell lysates were subjected to immunoblot using α-FLAG PAb. This figure presents the full-length blot and image of Fig. 5b.

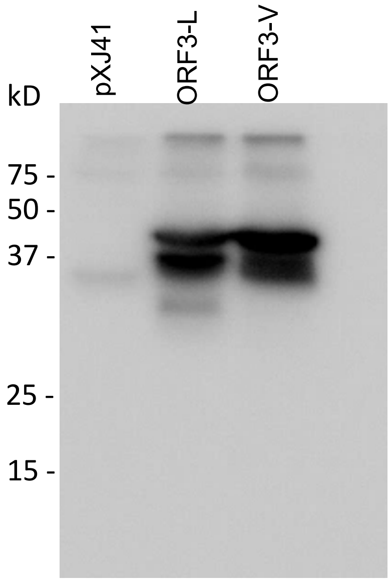

Supplement: Supplementary file 1 — Supplementary Information. [file 41598_2021_92941_MOESM1_ESM.pdf]
